# Optimizing pharmacologic treatment for neonatal opioid withdrawal syndrome (OPTimize NOW): a symptom-based dosing approach study protocol for a multi-center, cluster crossover design randomized controlled trial

**DOI:** 10.1186/s13063-025-09035-x

**Published:** 2025-08-27

**Authors:** Leslie W. Young, Denise C. Babineau, Abhik Das, Sara DeMauro, Walter K. Kraft, Scott Lorch, Michele C. Walsh, Stephanie Merhar, Lori A. Devlin, Eunice Kennedy Shriver National Institute of Child Health and Human Development Neonatal Research and the National Institutes of Health (NIH) Helping to End Addiction Long-term (HEAL) Initiative

**Affiliations:** 1 Department of Pediatrics, Larner College of Medicine at the University of Vermont, 111 Colchester Ave, Smith 5, Burlington, VT 05401 USA; 2 Social, Statistical, and Environmental Sciences Unit, RTI International, Durham, USA; 3 Department of Pediatrics, Children’s Hospital of Philadelphiaand, University of Pennsylvania Perelman School of Medicine , Philadelphia, USA; 4 Department of Pharmacology, Physiology and Cancer Biology, Thomas Jefferson University, Philadelphia, USA; 5 Eunice Kennedy Shriver National Institutes of Child Health and Human Development, Bethesda, MD USA; 6 Division of Neonatology, Department of Pediatrics, Perinatal Institute, Cincinnati Children’s Hospital Medical Center, University of Cincinnati, Cincinnati, USA; 7 Department of Pediatrics, University of Louisville School of Medicine, Louisville, USA

## Abstract

**Background:**

Opioid use and misuse during pregnancy rose from 1.5 to 6.5 per 1000 deliveries between 1999 and 2014 and continues as a significant public health concern. A fivefold increase in neonatal opioid withdrawal syndrome (NOWS) has accompanied the increase in opioid use. The Eating, Sleeping, Consoling care approach (ESC) has been shown to improve outcomes for infants with NOWS and is quickly becoming the standard of care for infants affected by opioid use disorder. Quality improvement initiatives following the implementation of ESC provide some evidence to suggest that symptom-based (i.e., as needed, PRN, just in time) dosing of opioid medications for infants with significant withdrawal may be an effective alternative to using a traditional scheduled opioid taper approach. These initiatives have shown reduced length of hospital stay and decreased postnatal opioid exposure when compared to scheduled opioid dosing for infants with NOWS who receive pharmacologic treatment. It is unknown if the findings from these quality improvement initiatives are generalizable, and little is known about the safety of this approach in a diverse population. The purpose of this manuscript is to describe the design and rationale for an ongoing study to evaluate the effect of symptom-based opioid dosing compared to traditional scheduled opioid taper on short-term outcomes for infants with NOWS.

**Methods/design:**

In this ongoing multi-center two-period cluster crossover randomized controlled trial, 24 sites within the USA were randomized at the site level into one of two sequences. Prior to randomization, sites were stratified by care approach used (ESC vs. usual care) and these strata were independently randomized. All study sites will provide care based on their random allocation. Data will be collected under waiver of consent for in-hospital and short-term outcomes for eligible infants. A minimum of 480 infants will be enrolled. We hypothesize that use of symptom-based dosing will safely reduce the length of time until infants with NOWS and at risk for pharmacological treatment are medically ready for discharge when compared to infants treated with a scheduled opioid taper.

**Discussion:**

This trial is uniquely and efficiently designed to establish the efficacy, safety, and generalizability of the symptom-based dosing approach to opioid treatment for NOWS.

**Trial registration:**

NCT05980260; registered July 27, 2023.

## Introduction

### Background and scientific rationale

Antenatal opioid exposure may result in neonatal opioid withdrawal syndrome (NOWS). Neonatal opioid withdrawal is characterized by dysfunction in 3 primary areas: (1) the central nervous system, (2) the autonomic nervous system, and (3) the gastrointestinal system. Signs of withdrawal include but are not limited to irritability, tremors, increased tone, hyperthermia, poor sleep, poor feeding, vomiting, and diarrhea. The natural history of NOWS is an initial increase in symptoms as placentally transferred opioids are cleared from the infant’s system. With time, withdrawal symptoms will eventually abate. The therapeutic focus is on the reduction of symptom severity, with a goal of ensuring normal infant growth and development and maternal-infant bonding. A focus on a function-based approach to care in addition to optimization of non-pharmacologic care, including a low stimulation environment, swaddling, clustered care and the provision of mother’s own milk coupled, when possible, with infant caregiver engagement to provide skin-to-skin care, and on demand breastfeeding has been shown to be effective as first-line treatment for infants with NOWS and is endorsed by the American Academy of Pediatrics (AAP) [1].

If non-pharmacologic care alone does not mitigate the severity of opioid withdrawal, pharmacologic treatment is indicated. The traditional approach to pharmacologic treatment for infants with NOWS is initiation and escalation of opioid treatment until symptoms are controlled followed by a slow opioid taper. This approach has been shown to be efficacious but is associated with extended opioid courses and prolonged hospitalization, both of which have been associated with suboptimal developmental outcomes. The use of *pro re nata* (PRN) or symptom-based dosing has been evaluated as a way to decrease postnatal pharmacologic exposure for infants with NOWS. Conceptually, a symptom-based approach would allow control of symptoms for infants who have severity of disease that borders on the usual trigger for the extended opioid treatment protocol. One to three doses of an opioid in such infants may be enough to control symptoms sufficiently and allow for non-pharmacologic therapies to be the primary mechanism for symptom control. This approach could avoid a prolonged course of opioids and weaning for a subset of infants with milder disease, but as an emerging approach it has not been validated. Quality improvement (QI) initiatives conducted in single centers and small regional collaboratives have demonstrated a decrease in the duration of pharmacologic treatment and the length of hospital stay with the use of a symptom-based approach as compared to an opioid taper approach. Improved short-term outcomes with a symptom-based dosing approach to pharmacologic treatment have been noted in infants assessed and managed with either the Eat, Sleep, and Console approach (ESC) or the Finnegan Neonatal Abstinence Assessment Tool (FNAST) [2–5]. In addition, the rate of readmission within 30 days of hospital discharge, a balancing measure used in the QI initiatives, was not increased with a symptom-based dosing approach when compared to a scheduled opioid taper.

QI initiatives have demonstrated a decrease in postnatal opioid exposure and the length of hospital stay with the use of a symptom-based dosing approach and have not found an increase in short-term adverse outcomes during the first month of life. As QI initiatives have been limited to single center or small regional collaboratives, and longer-term developmental outcomes have not been assessed, the efficacy, safety, and generalizability of a symptom-based dosing approach remains unknown and warrants evaluation with a multi-center randomized clinical trial.

#### Rational for selection of study intervention

The overarching hypothesis to be tested is if the use of a symptom-based dosing approach will lead to a reduction in the time until infants are medically ready for discharge when compared to a scheduled opioid taper approach. In the scheduled opioid taper approach, all patients who reach a symptom threshold will be treated with a study site’s usual pharmacologic treatment algorithm. Each algorithm is responsive to symptom severity to allow dosing escalation. All infants follow a protocolized weaning of opioid dose as prescribed by a study site’s pharmacologic treatment algorithm. The theoretic advantage of this approach is that infants with uncontrolled symptoms have a rapid dose escalation with rapid control of NOWS symptoms. The potential disadvantage is that infants are shunted into a treatment approach with denser opioid treatment than they may require for optimal symptom control, with an associated prolongation of hospital stay. The symptom-based approach allows for up to three individual doses in any 24-h period before beginning a scheduled dosing protocol. The theoretic advantage to this approach is that one to three single doses may be enough to control symptoms without requiring a fixed and prolonged regimen. A potential disadvantage is that this approach may delay the start of scheduled opioid dosing in some infants, which may ultimately increase the duration of hospitalization. It is also unclear if the number of infants treated, as defined as receipt of a single dose of opioid, will differ between the two groups. Quality improvement work suggests that clinicians may be more likely to give a single dose for infants with less severe symptoms rather than waiting until symptoms meet the typical thresholds for the initiation of an opioid taper, possibly leading to a greater proportion of infants treated at study sites using a symptom-based protocol.

The scheduled opioid taper approach is traditional management for infants with NOWS; it was developed empirically in the 1970 s and is widely used in clinical care. The symptom-based approach has emerged more recently as part of QI efforts to further tailor care specifically to the infant. The symptom-based approach has been employed entirely in the context of QI programs and efficacy has not been rigorously tested in a controlled fashion. For this reason, there is true equipoise between the two approaches, and both are used at high-quality academic and community hospitals.

Both approaches are currently used, with local variation in the type of primary opioid (methadone, morphine, and buprenorphine), starting dose, maximum opioid dose, weaning rate, cessation dose, and choice of secondary adjunctive medication for more severe cases (phenobarbital or clonidine). This study will employ a comparative effectiveness approach, in which each study site will continue to use its usual care for the primary opioid, dosing regimen, and secondary medication. The rationale for this pragmatic approach is that the overarching question of scheduled or symptom-based dosing is of prime importance and has a larger impact than any site-specific regimen. As all protocols are responsive to individual patient symptoms, the impact of minor differences between study sites will likely not impact the overall comparison. The use of a crossover design will minimize the inter-site differences on the primary comparison. Lastly, the comparative effectiveness paradigm will foster site acceptance and recruit a wider swath of hospital types and patient populations, enhancing the generalizability of the study findings.

## Study hypothesis and objectives

### Primary hypothesis

The length of time from birth until medically ready for discharge will be shorter in infants with NOWS who are ≥ 36 weeks’ gestation, at risk for pharmacologic treatment, and managed for NOWS with a symptom-based dosing approach than in infants with NOWS who are ≥ 36 weeks’ gestation, at risk for pharmacologic treatment, and managed for NOWS with a scheduled opioid taper approach.

The primary hypothesis will be separately tested in two populations:

1. Infants who were assessed and managed with ESC (primary objective)
2. Infants who were assessed and managed with FNAST (secondary objective #1)

### Primary objective

To compare the length of time from birth until medically ready for discharge between infants with NOWS who are ≥ 36 weeks’ gestation, at risk for pharmacologic treatment, who are assessed and managed with ESC, and managed for NOWS with either a symptom-based dosing approach or a scheduled opioid taper approach.

### Secondary hypothesis

All enrolled infants:

1. To compare the length of time from birth until medically ready for discharge between infants with NOWS who are assessed and managed with FNAST and managed for NOWS with either a symptom-based dosing approach or a scheduled opioid taper approach
2. To compare the receipt of pharmacologic treatment for NOWS between infants managed for NOWS with either a symptom-based dosing approach or a scheduled opioid taper approach
3. To compare the length of hospital stay between infants managed for NOWS with either a symptom-based dosing approach or a scheduled opioid taper approach
4. To compare safety outcomes during the first 3 months of life between infants with NOWS who were managed with either a symptom-based dosing approach or a scheduled opioid taper approach

All enrolled infants who were pharmacologically treated:

5. To compare the total number of opioid doses administered between infants with NOWS who were pharmacologically treated with either a symptom-based dosing approach or a scheduled opioid taper approach
6. To compare the receipt of secondary medications between infants with NOWS who were pharmacologically treated with either a symptom-based dosing approach or a scheduled opioid taper approach
7. To evaluate the proportion of infants with NOWS who were initially pharmacologically treated with a symptom-based dosing approach but transitioned to a scheduled opioid taper due to persistent signs of withdrawal
8. To evaluate the proportion of infants with NOWS who were initially pharmacologically treated with a scheduled opioid taper but stopped treatment due to excessive sedation or respiratory depression
9. To compare safety outcomes during the first 3 months of life between infants with NOWS who were pharmacologically treated with either a symptom-based dosing approach or a scheduled opioid taper approach

## Study design

### Description of study design

**Fig. 1:**
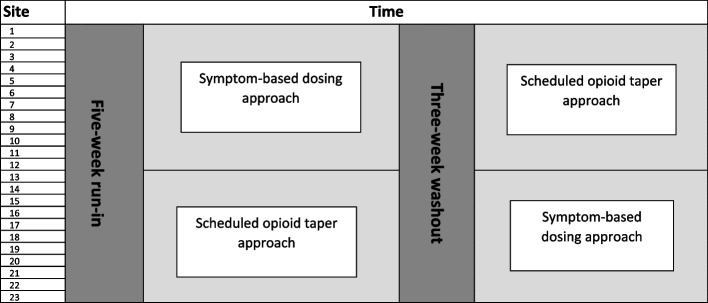
Two-period crossover study design

The study design is a two-period cluster crossover study design which groups study sites into clusters and randomly assigns each cluster to one of two sequences (i.e., 5-week run-in followed by scheduled opioid taper approach for 5 months (period 1) followed by a 3-week washout followed by symptom-based dosing approach for 5 months (period 2) or 5-week run-in followed by symptom-based dosing approach for 5 months (period 1) followed by a 3-week washout followed by scheduled opioid taper approach for 5 months (period 2)) (see Fig. 1). The duration of each period is expected to be 5 months; this length may need to be modified to ensure sample size is met in Finnegan and ESC groups. Each study site will use the same assessment and management approach (i.e., FNAST or ESC) and will not vary their preferred opioid for primary treatment or alter their pharmacologic treatment algorithm throughout the entire duration of the study.

#### Study intervention

**Fig. 2:**
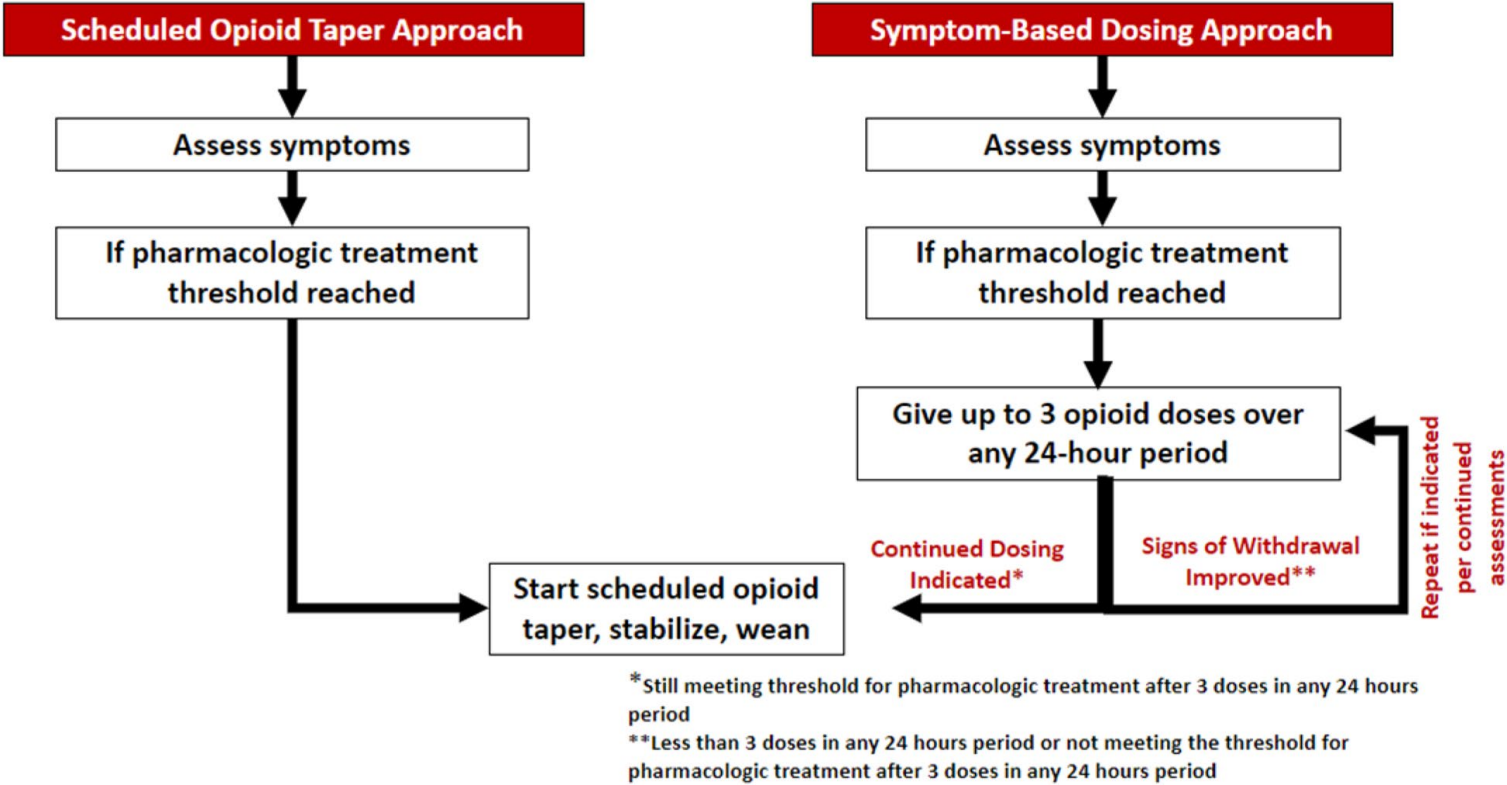
Dosing approach graphic

The interventions for this study, the scheduled opioid taper approach and the symptom-based dosing approach (see Fig. 2), were chosen as they are both being utilized as a clinical approach to the pharmacologic treatment for infants with NOWS, yet no generalizable evidence is available to support efficacy or safety of one approach over the other.

##### Intervention description

Throughout this study, all study sites will utilize their assigned dosing approach for the management of all infants with NOWS who meet the threshold for the initiation of opioid treatment as defined by each study site’s pharmacologic treatment algorithm and will transition their dosing approach per their randomized allocation. All study sites will adhere to minimum initial and maximum daily doses of opioids.

Scheduled opioid taper approach:

- During this approach to care, all enrolled infants with NOWS at the study site will be treated with the study site’s usual scheduled opioid taper approach, as detailed in each study site’s treatment algorithm, if they meet the study site’s withdrawal threshold for pharmacologic treatment.
- Each study site will utilize their preferred opioid (i.e., morphine, methadone, buprenorphine) and their usual scheduled opioid taper algorithm, as long as the study site’s algorithm meets the parameters established by the protocol study team.
- Weaning of opioid medications will be per the study site’s scheduled taper and hospital discharge will be per the study site’s usual practice.
- All infants will be monitored off opioid medications for worsening signs of withdrawal prior to hospital discharge as per the study site’s usual clinical practice.

Symptom-based dosing approach:

- During this approach to care, all enrolled infants with NOWS at the study site will be treated with the symptom-based dosing approach if they meet the withdrawal threshold for pharmacologic treatment.
- Each study site will utilize their preferred opioid (i.e., morphine, methadone, buprenorphine) and a symptom-based dosing approach to pharmacologic treatment.
- Infants with NOWS may receive up to 3 doses of the study site’s preferred opioid during any 24-h period to treat signs of withdrawal once the threshold for pharmacologic intervention is met.
- The opioid dose used during this approach is the initial opioid dose on each site’s pharmacologic treatment algorithm. The minimum duration between symptom-based doses will be determined by the opioid type used at the study site. Examples are provided in the dosing approach graphic below. If an infant meets the threshold for pharmacologic treatment at the minimum dosing interval for the type of opioid used at the site (i.e., short interval dosing) across two assessment periods, the infant will move to the schedule opioid algorithm at the first up titration dose (with the third dose of opioid medication). The need for recurrent dosing at the minimum duration threshold suggests that the severity of opioid withdrawal is unlikely to be well controlled with symptom-based dosing and early transition to scheduled opioid treatment would better support the physiologic needs of the infant.
- If a 4th dose is required within any 24-h period, the study site will move to scheduled opioid treatment per the algorithm used at the study site to complete the infant’s pharmacologic treatment. The 4th dose would include the first up titration of the study site’s scheduled dosing protocol.
- If opioid dosing is scheduled, weaning of opioid medications will be per the study site’s scheduled taper and hospital discharge will be per the study site’s usual practice.
- During this approach, it is recommended that all infants be monitored off opioid medication for worsening signs of withdrawal for ≥ 48 h prior to hospital discharge.

##### Dosing parameters

*Opioid therapy*

Pharmacologic treatment regimens vary greatly among clinical sites treating NOWS [6, 7]. While morphine remains the most commonly used agent [8], the individual drugs, initial and maximum doses, and weaning rates are not standardized. In addition, some sites use a fixed percentage up titration rate independent of symptom scores, while others use a non-weight-based dose titration rate driven by score severity. Neither of these two approaches have been standardized, nor is there evidence of the superiority of either approach [9]. The dynamic nature of dosing based upon a disease severity score provides significant protection against excess dosing and respiratory depression in the highly monitored settings in which NOWS is treated, particularly during the intensive up titration phase until capture of scores is obtained. Excess sedation will be evident before respiratory depression emerges. Study sites all have well developed internal protocols for dosing that have evolved over many years, as well as robust monitoring procedures with discretion of caregivers to hold doses based upon clinical presentation of the infants. These make AEs of excess sedation from opioids exceeding uncommon during routine clinical care. There have been pharmacokinetic investigations to explore dose optimization [10–14], but none has been extensively tested and validated. These factors and the use of a crossover design favor the use of site-specific regimens in a comparative effectiveness approach rather than a uniform dosing approach. Minimum dosing intervals (i.e., time between doses) for each opioid type have been developed by the study team and should be considered when developing the symptom-based dosing algorithm at each study site. All study sites will adhere to the following broad dosing parameters (see Table 1).

**Table 1:** Opioid therapy dosing parameters

	Morphine	Methadone	Buprenorphine
Minimum initial dose	0.05 mg/kg	0.1 mg/kg	4.5 mcg/kg
Maximum daily dose	1.6 mg/kg/day	0.6 mg/kg/day	75 mcg/kg/day

*Adjunctive secondary and tertiary medications*

An evidence-based choice of optimal secondary medication is also not defined in the literature. Though doses are largely empirically derived, extrapolated from adults, or in the case of phenobarbital extrapolated from use in seizure control, there is a greater convergence upon a more standardized dose regimen than with opioids. All study sites will adhere to the following broad dosing parameters (Table 2).

**Table 2:** Secondary and tertiary medication dosing parameters [15]

	Phenobarbital	Clonidine
Maximum loading dose	20 mg/kg	N/A
Minimum initial dose	1.25 mg/kg/day	0.625 mcg/kg
Maximum daily dose	5 mg/kg/day	24 mcg/kg/day

All enrolled infants will be assessed at 3 months of age for the use of nonscheduled medical care which includes acute/urgent care visits, emergency room visits, and hospital readmissions. This assessment will include a review of the medical records which will include data from the hospital in which the infant was born, and any additional information that can be assessed through linked medical records.

All enrolled infants will be assessed for seizures and death until hospital discharge.

All enrolled infants will also be assessed for non-accidental trauma or death at 3 months of age. These assessments will include a review of the medical records including data from the hospital in which the infant was born, and any additional information that can be assessed through linked medical records, in addition to a review of public facing media.

### Primary endpoint

Time from birth until infant meets criteria for medically ready for discharge (days). An infant is considered medically ready for discharge when they are discharged by the medical provider or when they meet both of the following criteria:

- ≥96 h of age
- ≥48 h since last dose of opioid treatment (i.e., morphine, methadone, or buprenorphine)

### Secondary endpoints

All enrolled infants:

- Receipt of pharmacologic treatment (yes/no)
- Time from birth until hospital discharge (days)
- Inpatient composite safety outcome (present/absent)
  - ◦ Seizures
  - ◦ Excessive weight loss more than 15% from birthweight
- Inpatient composite critical safety outcome (present/absent)
  - ◦ Non-accidental trauma
  - ◦ Death
- Outpatient composite safety outcome (present/absent)
  - ◦ Acute/urgent care or emergency room visits
  - ◦ Hospital readmissions
- Outpatient composite critical safety outcome (present/absent)
  - ◦ Non-accidental trauma
  - ◦ Death

Among all enrolled infants who were pharmacologically treated:

- Number of doses of primary medication administered (count)
- Receipt of secondary medications (yes/no)
- Inpatient composite safety outcome (present/absent)
  - ◦ Seizures
  - ◦ Excessive weight loss more than 15% from birthweight
- Inpatient composite critical safety outcome (present/absent)
  - ◦ Non-accidental trauma
  - ◦ Death
- Outpatient composite safety outcome (present/absent)
  - ◦ Acute/urgent care or emergency room visits
  - ◦ Hospital readmissions
- Outpatient composite critical safety outcome (present/absent)
  - ◦ Non-accidental trauma
  - ◦ Death

Among all enrolled infants who were initially pharmacologically treated with a symptom-based dosing approach:

- Transition to an opioid taper due to persistent signs of withdrawal (yes/no)

Among all enrolled infants who were initially pharmacologically treated with a scheduled opioid taper approach:

- Cessation of opioid treatment due to excessive sedation or respiratory depression (yes/no)

### Stratification, randomization, and blinding/masking

Each study site (i.e., cluster) will be randomized in a 1:1 allocation ratio to one of two sequences, following a 5-week run-in:

- Scheduled opioid taper approach for 5 months (period 1) followed by a 3-week washout followed by symptom-based dosing approach for 5 months (period 2)
- Symptom-based dosing approach for 5 months (period 1) followed by a 3-week washout followed by scheduled opioid taper approach for 5 months (period 2)

The randomization scheme will be stratified by the assessment and management approach used at each study site (i.e., FNAST or ESC). Study sites will be notified of their allocated sequence following randomization.

Due to the nature of the study intervention, the protocol study team, site PI, site study team, and participants will be unblinded to the assigned study intervention.

## Participant selection

### Study setting

At least 24 sites from varying geographic areas in the USA will be selected for participation in this study and will include community hospitals, academic hospitals, and regional pediatric referral centers.

### Inclusion criteria

Sites who meet all of the following criteria are eligible to participate in this study:

1. The site is willing and able to transition to a symptom-based dosing approach at a randomly allocated time and is willing and able to transition from a symptom-based dosing approach to a scheduled opioid taper approach at a randomly allocated time.
2. The site uses either the Eat, Sleep, and Console care approach or the Finnegan Neonatal Abstinence Scoring Tool or modification thereof to assess infants with NOWS and will continue to use the same assessment and management approach throughout the study.
3. The site provides scheduled opioid treatment with morphine, methadone, or buprenorphine for infants with NOWS and has an established algorithm for a scheduled pharmacologic taper prior to study initiation. The site agrees to maintain the type of opioid(s) used and use the study approved treatment algorithms throughout the study.

Individuals who meet all the following criteria are eligible for enrollment as study participants:

1. The infant is ≥ 36 weeks’ gestation.
2. The infant had antenatal opioid exposure identified by at least one of the following:
  ◦ History of maternal opioid use during pregnancy
  ◦ Positive maternal toxicology screen for opioids during the second or third trimester of pregnancy
  ◦ Positive infant toxicology screen for opioids during the initial hospital stay
3. The infant is being assessed and managed for NOWS at an eligible study site.
4. The infant is at risk for pharmacologic treatment for NOWS defined by either of the following:
  ◦ At least 1 score ≥ 8 if assessed and managed with FNAST or modification thereof
  ◦ At least 1 yes if assessed and managed with the ESC care approach

### Exclusion criteria

Sites who meet any of these criteria are not eligible to participate in this study:

1. The site manages less than 5 infants who receive pharmacologic treatment for NOWS annually.
2. The site routinely discharges/transfers infants from the hospital on opioid treatment (i.e., morphine, methadone, or buprenorphine). Routine is defined as ≥ 10% of infants who receive opioid treatment for NOWS at the time of hospital discharge.
3. The site routinely uses more than one opioid type for the pharmacologic treatment of infants with NOWS. Routine is defined as ≥ 10% of infants who receive more than one opioid for the treatment of NOWS.

Individuals who meet any of these criteria are not eligible for enrollment as study participants:

1. The infant has major birth defect(s).
2. The infant has neonatal encephalopathy (inclusive of hypoxic ischemic encephalopathy), a metabolic disorder, stroke, intracranial hemorrhage, or meningitis diagnosed prior to the initiation of pharmacologic treatment.
3. The infant is receiving respiratory support (any positive pressure or oxygen therapy) at 48 h of age.
4. The infant has undergone major surgical intervention prior to or at 48 h of age.
5. The infant has postnatal opioid exposure prior to the initiation of pharmacologic treatment for NOWS.
6. The infant was outborn and pharmacologic treatment was initiated at the transferring hospital.
7. The infant is assessed for eligibility during the study site’s 3-week washout.

### Recruitment

The protocol study team will screen interested sites for eligibility and will randomize eligible sites into one of two arms (see Fig. 1). With the proposed study design, all infants with NOWS cared for at each study site will be managed using the dosing approach assigned to the study site during one of the two study periods. There will be a 5-week run-in prior to the first study period. There will be a 3-week washout between the end of the first study period and start of the second study period where no infants will be enrolled. Study sites will transition their pharmacologic treatment approach per their randomized allocation during the 3-week wash out period. The inpatient clinical team will assess infants with antenatal opioid exposure and establish the diagnosis of NOWS. The initiation of clinical management for infants with NOWS will not be impacted by the study intervention. Non-pharmacologic care will be considered first-line treatment for all infants with NOWS at study sites and will be implemented and maximized per each study site’s usual approach. The site study team will identify potential participants based on their eligibility following review of the medical record after delivery.

Site recruitment and retention: The protocol study team began to optimize the potential for recruitment during initial protocol development through an assessment of potential study sites’ willingness to participate in and enthusiasm for various study designs. The study design chosen for this protocol incorporates feedback from potential sites. During the site assessment process, the protocol study team will expect each site to commit in writing to the site’s participation in and completion of the study with maintenance of the site’s allocated study intervention for the duration of the study. The protocol study team will facilitate retention of study sites through the focused allocation of funds to support participation, assessment of needs, provision of support, and troubleshooting at each study site, as needed.

## Known and potential risks and benefits

### Potential risks

Under the proposed study design, all infants managed for NOWS at a study site will receive care consistent with the pharmacologic approach assigned by the protocol study team. Sites throughout the country are currently using both dosing approaches described in this study. Use of either pharmacologic approach will not expose infants in this study to risk beyond that of usual/accepted clinical care. This is minimal risk research. Clinical experience and pharmacokinetic investigations provide assurance of the safety of three symptom-driven administrations of a fixed low dose every 1 to 2 h for morphine, methadone, and buprenorphine. Systemic drug exposure of morphine, methadone, or buprenorphine will not extend beyond that seen in usual clinical care for NOWS [10, 16–18].

Involvement in the study will not increase the risk to the family of legal ramifications associated with the in utero opioid exposure of their infants, as only infants who are diagnosed with and being actively managed for NOWS will be screened for enrollment in the study. There will be no additional toxicology screening (maternal or infant) performed beyond what medical professionals would typically obtain as part of usual institutional care at the study site. Thus, there will be no additional information garnered with respect to substance use during pregnancy due to involvement in the study. As an NIH-funded investigation, this study has the default protections of a Certificate of Confidentiality for all sensitive patient information.

Children in the study and their mothers will have medical records reviewed by study personnel. This is a small risk of loss of confidentiality of medical record information. All data files will be coded in a manner that makes identification of the subjects unlikely. Subjects’ identification codes are simply their numerical enrollment in the study and thus do not lend themselves to inadvertent or unauthorized identification of subjects. Transmission of electronic records will be over a secure encrypted connection and will not contain Protected Health Information (PHI). Only coded data will be transmitted and maintained at the DCC.

### Potential benefits

There is equipoise about the optimal dosing approach, and using a cluster crossover design, all infants at a study site will receive the same dosing approach during a set study period. There is empirical evidence that infants enrolled in clinical trials have better outcomes than similar infants treated outside of a clinical trial [19], and that the outcomes of all infants are better at centers that routinely conduct clinical trials. Within the context of NOWS therapeutics, there is evidence of the benefit of a uniform and protocol-driven approach to therapeutics which will be a key element of this clinical trial. Thus, it is likely that the care of infants at all study sites will be improved, regardless of the specific study period during which they are enrolled [20].

## Study procedures and data collection

### Study assessments/evaluations

A summary of complete study procedures is detailed in the study schedule. Details of each assessment are provided below.

#### Among all screened infants

Maternal race and ethnicity will be assessed on all screened infants.

#### Among all enrolled infants

The following data, where available, will be collected on all enrolled infants:

- Study participant demographics including sex, date and time of birth, gestational age at birth, birthweight, length at birth, and head circumference at birth
- Maternal date of birth, marital status, level of education, medical insurance, and zip code (which will be converted to a rural–urban community area (RUCA) code for analysis), medical history during pregnancy and at delivery, and exposures during pregnancy
- Neonatal toxicology after birth
- Receipt of pharmacologic treatment for NOWS
- Assessment and management approach, including ESC or FNAST assessments/scores
- Daily weight from birth to hospital discharge
- Location of care in the hospital
- Date and time of hospital discharge
- Incidence of death until hospital discharge
- Safety outcomes (see sections on collection and reporting of adverse events)

#### Among all enrolled infants who were pharmacologically treated

The following data will be collected on all enrolled infants who were pharmacologically treated during the initial hospitalization following birth:

- Each type and dose of opioid treatment (morphine, methadone, buprenorphine) prior to hospital discharge
- Each type and dose of adjuvant therapy (clonidine or phenobarbital) prior to hospital discharge
- Daily feeding information, including type of enteral feedings including formula type, route of administration, and caloric content, from birth to hospital discharge

### SPIRIT figure: OPTimize NOW study events


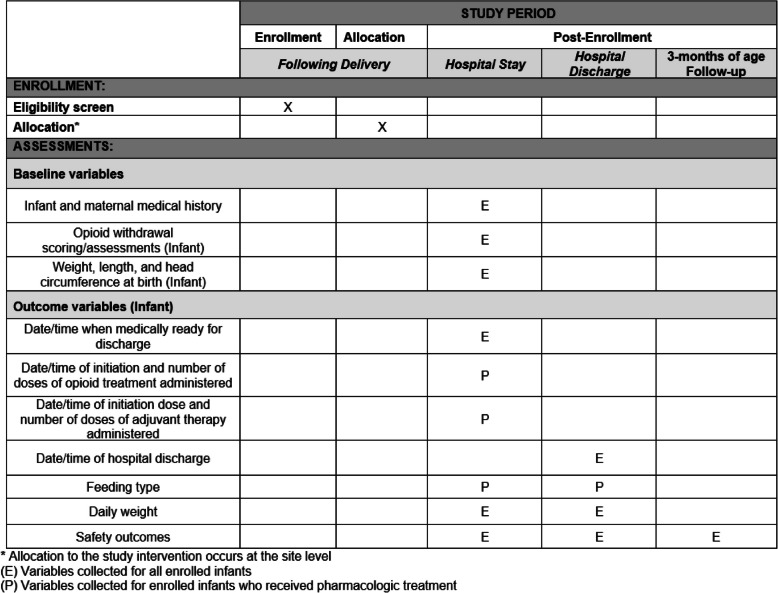


### Data collection methods

Data collected will be from the infant and maternal medical records, including transfer records. All clinical data collected from this study will be extracted directly from the electronic health records or source documents. There will be no interactions with, nor will there be any information collected directly from, the mother, the infant, or the treating clinician.

The site research team will assess the outpatient composite safety outcome as well as the outpatient critical safety outcome through review of the medical records (including the study site’s primary and any linked electronic medical record systems) and media review (public facing) for all enrolled infants at approximately 3 months of age.

## Criteria for participant and study completion and premature discontinuation of study intervention and study termination

### Participant completion

Participant completion will be at 3 months of age.

### Participant stopping rules and withdrawal criteria

Participants may be prematurely terminated from the study for the following reasons:

1. The participant dies.
2. The PI no longer believes participation is in the best interest of the participant.
3. If the family requests that data not be collected on their infant.

### Study stopping rules

The study will be temporarily paused to review safety data if any of the following study stopping rules are met:

- More than 1% of the enrolled infants have an inpatient seizure, as assessed on a rolling basis, and the total number of cases equaling 7 or more.
- More than 35% of the enrolled infants who were pharmacologically treated have an outpatient acute/urgent care or emergency room visit, as assessed on a rolling basis, and the total number of cases equaling 252 or more.
- More than 10% of the enrolled infants who were pharmacologically treated have an outpatient hospital readmission, as assessed on a rolling basis, and the total number of cases equaling 72 or more.

## Monitoring

### Data monitoring

To assure the quality of the data collected, the protocol study team will provide training specific to accuracy of data acquisition for the site study team at each study site. The protocol study team will design CRFs, which a subset of study sites will subsequently pilot to minimize the potential for errors. Additionally, the protocol study team will allocate sufficient funds to allow for quality data collection. The site study team will re-abstract a subsample of their own charts and assess the error rate. Re-abstraction will focus on critical data elements related to the primary and secondary objectives of the protocol. The protocol study team will base the number of charts a study site re-abstracts, for each study period, on the number of patients enrolled at the mid-point in the study period as shown below:

**Table:** 

No. of patients enrolled during the study period	No. of charts to be re-abstracted
0	0
1–14	1
15–24	2
25–34	3
35–44	4
45–54	5

The DCC will provide study sites with the randomly selected subject IDs for re-abstraction. The site study team will identify an independent site quality control (QC) abstractor who will re-abstract and enter data into the electronic data capture system (EDC) only for the QC process and will not abstract study data while QC activities are taking place. The DCC will generate a discrepancy report comparing study data abstracted by the study site with the source information abstracted by the independent site QC abstractor. The DCC will hold a QC Review Meeting with the site study team, to include the independent site QC abstractor, research coordinator, and original site abstractor(s) to review the discrepancies and identify errors. Together they will discuss and document the corrective action for each error identified. The DCC will create manual queries in the EDC to make any necessary corrections to the data that QC review members identify. The protocol study team will provide study sites that have an error rate above the predefined threshold with additional training, a site-specific assessment of the data collection process, and suggestions for process improvement. The protocol study team will track study sites by their error rates. The protocol study team will share practices of those study sites with exceptionally low error rates with study sites working to improve their own process. The protocol study team will review error rates and re-abstraction data during monthly team calls. If errors exceed the predefined threshold on 2 consecutive reviews, a remediation plan will be requested and shared with the study sponsor. Study sites that have an error rate above the predefined threshold will receive additional training, a site-specific assessment of the data collection process, and suggestions for process improvement.

#### Interim analyses

One interim analysis of efficacy of the primary endpoint (as well as associated safety data) will be performed when the assessment of the primary endpoint is completed in approximately 50% of enrolled infants with NOWS. The DSMB will review the results of the interim analysis. Based on pre-specified study stopping rules outlined in the SAP, the DSMB will provide a recommendation to the protocol study team and study sponsor regarding stopping the study early due to evidence of efficacy or continuing enrollment as planned.

### Adverse events

#### Definition of adverse events and serious adverse events

AE: AE means any untoward medical occurrence associated with the use of an intervention in humans, whether or not considered study intervention related.

AEs of special interest (AESI): AESIs will include seizures, excessive weight loss (more than 15% from birthweight), and respiratory insufficiency that occur during the initial hospitalization, after study enrollment, through hospital discharge.

Serious adverse event (SAE): An AE is considered “serious” if, in the view of either the site PI or the study sponsor, it results in any of the following outcomes:

1. Death
2. Life-threatening AE (life-threatening means that the study participant was, in the opinion of the site PI or study sponsor, at immediate risk of death from the reaction as it occurred and required intervention)
3. Persistent or significant incapacity or substantial disruption of the ability to conduct normal life functions
4. Inpatient hospitalization or prolongation of existing hospitalization
5. Important medical event that may not result in one of the above outcomes but may jeopardize the health of the study participant or require medical or surgical intervention to prevent 1 of the outcomes listed in the above definition of serious event

#### Classification of an adverse event

##### Severity of event

For AEs, the site PI will use the following guidelines to describe severity.

- Mild: Events require minimal or no treatment and do not interfere with the participant’s daily activities.
- Moderate: Events result in a low level of inconvenience or concern with the therapeutic measures. Moderate events may cause some interference with functioning.
- Severe: Events interrupt a participant’s usual daily activity and may require systemic drug therapy or other treatment. Severe events are usually potentially life-threatening or incapacitating. Of note, the term “severe” does not necessarily equate to “serious.”

##### Relationship to study intervention

The site study team will grade the degree of certainty about causality by using the below categories:

- Definitely related—There is a definite temporal and logical/medical relationship to a study procedure.
- Probably related—There is a strong temporal relationship to study procedure and another cause of event is unlikely or significantly less likely.
- Possibly related—There is a strong temporal relationship to the study procedure and another cause of event is equally or less likely compared to the potential relationship to study procedure.
- Unlikely related—There is little or no temporal relationship to the study procedure and/or a more likely other cause of event exists.
- Not related—There is not a reasonable possibility that the study procedures caused the AE, there is no temporal relationship between the study procedures and AE onset, or an alternate etiology has been established.

#### Collection of adverse event assessment and follow-up

For this study, the site study team will collect adverse event data on all enrolled infants:

- From enrollment to hospital discharge:
  - ◦ Seizure (AESI)
  - ◦ Respiratory insufficiency (AESI)
  - ◦ Weight loss > 15% of birthweight (AESI)
  - ◦ Death (SAE)
  - ◦ Life-threatening AE (life-threatening means that the study participant was, in the opinion of the site PI or study sponsor, at immediate risk of death from the reaction as it occurred and required intervention) (SAE)
  - ◦ Persistent or significant incapacity or substantial disruption of the ability to conduct normal life functions (SAE)
  - ◦ Inpatient hospitalization or prolongation of existing hospitalization (SAE)
  - ◦ Important medical event that may not result in one of the above outcomes but may jeopardize the health of the study participant or require medical or surgical intervention to prevent 1 of the outcomes listed in the above definition of serious event (SAE)
- From discharge through 3 months of age:
  - ◦ Death (SAE)
  - ◦ Life-threatening AE (life-threatening means that the study participant was, in the opinion of the site PI or study sponsor, at immediate risk of death from the reaction as it occurred and required intervention) (SAE)
  - ◦ Persistent or significant incapacity or substantial disruption of the ability to conduct normal life functions (SAE)
  - ◦ Inpatient hospitalization or prolongation of existing hospitalization (SAE)
  - ◦ Important medical event that may not result in one of the above outcomes but may jeopardize the health of the study participant or require medical or surgical intervention to prevent 1 of the outcomes listed in the above definition of serious event (SAE)

The occurrence of an AESI or SAE may come to the attention of site study team during the hospital stay, by the clinical team, or upon reviewing data. The site study team will capture all AESIs or SAEs on the appropriate CRF. Information to be collected includes event description, date/time of onset, date/time of resolution, clinician’s assessment of severity, relationship to study intervention, and time of resolution/stabilization of the AE. The site study team must follow all AESIs until the AESI meets one of the following criteria: resolution, the condition stabilizes, the AESI is otherwise explained or is judged by the protocol study team to be no longer clinically significant, or the participant is lost to follow-up.

Each of the site PIs (or designee) is responsible for ensuring that the completed AE form along with additional laboratory findings or other documentation required to describe the event is submitted to the DCC as specified in the manual of procedures. The study participant records are identified by study ID number and all other identifying information is stripped from the forms.

#### Reporting of adverse events

Cumulative AE data are reviewed by the DSMB at scheduled meetings during the study. The DCC will prepare reports of the study for review by the DSMB. Any AEs may be reported to the DSMB at other times at the request of NICHD.

The DCC will report safety events to the sIRB as needed.

### Protocol deviations

The DCC will monitor protocol deviations per study site in relation to the number of participants enrolled. All study sites will receive re-education via regularly scheduled teleconferences to help other study sites prevent similar deviations. If a particular deviation is recurrent at one study site or across the study sites, the DCC may implement operational tools, such as additional reminders, source document worksheets, and/or checklists, to reduce the likelihood of deviations. The DCC will review protocol deviations throughout the study, and it may schedule additional on-site visits, as needed, to review regulatory documents, data points, key issues, etc. or to retrain site study team to improve processes and provide additional education.

#### Protocol deviations definitions

Protocol deviation—The site PI and site study team will conduct the study in accordance with the protocol; no deviations from the protocol are permitted. Any change, divergence, or departure from the study design or procedures constitutes a protocol deviation. As a result of any deviation, corrective actions will be developed by the study site and implemented promptly.

Major protocol deviation (protocol violation)—A protocol violation is a deviation from the sIRB approved protocol that may affect the subject’s rights, safety, or well-being and/or the completeness, accuracy, and reliability of the study data. In addition, protocol violations include willful or knowing breaches of human subject protection regulations, or policies, any action that is inconsistent with the NIH Human Research Protection Program’s research, medical, and ethical principles, and a serious or continuing noncompliance with federal, state, local, or institutional human subject protection regulations, policies, or procedures.

#### Reporting and management of protocol deviations

The site PI has the responsibility to identify, document, and report protocol deviations as directed by the study sponsor. However, protocol deviations may also be identified during site monitoring visits or during other forms of study conduct review.

## Ethics and dissemination

### Research ethics approval and IND exempt status

A sIRB will be used for all study sites.

#### Waiver of IND

The study will be exempt from IND based upon the following criteria, all of which are true in this investigation [21].

- The drug product is lawfully marketed in the USA.
- The investigation is not intended to be reported to the US Food and Drug Administration as a well-controlled study in support of a new indication and there is no intent to use it to support any other significant change in the labeling of the drug.
- The investigation is not intended to support a significant change in the advertising for the drug.
- The investigation does not involve a route of administration, dose, patient population, or other factor that significantly increases the risk (or decreases the acceptability of the risk) associated with the use of the drug product (21 CFR 312.2(b)(1)(iii)).
- The investigation is conducted in compliance with the requirements for review by an IRB (21 CFR part 56) and with the requirements for informed consent (21 CFR part 50).
- The investigation is conducted in compliance with the requirements of § 312.7 (i.e., the investigation is not intended to promote or commercialize the drug product).

The clinical trial employs a pragmatic approach in which study sites will largely use their preferred opioid (morphine, methadone, buprenorphine) in usual care excipients and at doses which they use in the clinical care context. The change of dosing approach is not expected to have a different risk profile based upon treatment allocation.

### Consent or assent

#### Waiver of consent for in-hospital participation

The use of a cluster crossover design for this study will allow for randomization of the study intervention at the level of the study site. Study sites will adhere to their assigned approach for the pharmacologic treatment of all infants with NOWS cared for at their site throughout the study. Maintaining a generalizable study population will be essential to ensure that the results of this study will inform clinical care. Variation in antenatal exposure, from consistent daily exposure to medical treatment of opioid use disorder to active use of illicit opioids, complicates neonatal opioid withdrawal and may impact an infant’s response to the approach to pharmacologic treatment. As such, we request a waiver of consent from the sIRB for this study to minimize the risk of biasing the sample towards stable maternal/infant dyads with less severe opioid dependency and comorbidities.

A cluster-randomized clinical trial may proceed without individual consent if Common Rule conditions for a waiver of consent are satisfied, and participants (or legally authorized representatives) are provided with a description of the study intervention to which their cluster has been randomized. As stated in Code of Federal Regulations [45 CFR 46.116 (d)], an sIRB may approve a consent procedure that does not include, or that alters, some or all of the elements of informed consent set forth in this section, or waive the requirements to obtain informed consent, provided the sIRB finds and documents that all of the following conditions are met:

1. The research involves no more than minimal risk to the participants.
2. The waiver or alteration will not adversely affect the rights and welfare of the participants.
3. The research could not practicably be carried out without the waiver or alteration.
4. Whenever appropriate, the study team will provide participants with additional pertinent information after participation.

The justification for a waiver of informed consent from caregiver(s) for the previously outlined study objectives meets the above criteria as follows:

1. The research involves no more than minimal risk to the participants.
  Both approaches to pharmacologic treatment (i.e., the symptom-based dosing approach and scheduled opioid taper approach) are currently used at sites across the country, and the optimal approach to pharmacologic treatment for infants with NOWS is unknown. The protocol study team has utilized recommendations from the AAP to define the primary outcome to further facilitate safe discharge practices for all infants with NOWS. Undertreatment and overtreatment will be minimized by the study parameters developed by the protocol study team and a DSMB will be engaged to identify any risk concerns throughout the study. The usual approach to management of NOWS is protocol-driven based upon a site level therapeutic approach. Dosing approach (symptom versus scheduled) is not decided at the level of the individual patient nor at the discretion of an individual practitioner. Thus, the role of removing individual physician discretion for specific infants, a commonly cited criteria against waiver of consent, is not an issue in NOWS in which dosing approach is determined at the facility level. This clinical trial will employ two widely accepted dosing approaches. In addition, the treatment of NOWS in the inpatient setting is extremely safe regardless of the opioid employed or dosing regimen. This is due to drug dosing that is directly tied to symptom scores every 4 h, signs of excess sedation being evident much before respiratory depression, and careful respiratory monitoring in an inpatient setting.
2. The waiver or alteration will not adversely affect the rights and welfare of the participants.
  As the best pharmacologic management for infants with NOWS is unknown, there is no universally accepted standard approach, and both management strategies are currently being used at sites across the country. Therefore, participants receiving care via either approach to pharmacologic treatment should not have their rights and welfare adversely affected.
3. The site research team could not practicably carry out this trial without the waiver or alteration.
  Recent clinical trials in NOWS for which individual patient consent to an altered therapeutic regimen (NCT04214834, 04104646) have encountered a patient consent rate of only 20–30% of those eligible. Though the patient parental characteristics of those who have declined participation cannot be collected in systematic fashion, clinical trialists at these sites have noted those declining tend to have more polysubstance abuse, have higher use of prescribed benzodiazepines and psychotropics, are less likely to be in an outpatient opioid use disorder program, and often have impulse control as part of a comorbid psychiatric diagnosis. Most of these factors have been linked to worse outcomes (as measured by symptom scores and length of treatment for infants). Large (for NOWS) randomized trials with individual level consent have all tended to have enrolled mothers with less of the risk factors listed above [22–24]. The feasibility and generalizability of this trial would be limited if obtaining informed consent were required. It is highly possible that infants with antenatal exposure to illicit or misused opioids may respond differently to symptom-based dosing approach than infants with antenatal exposure to medications for opioid use disorder (MOUD). The goal of this research is to identify a generalizable approach to pharmacologic treatment for infants with NOWS. Enrolling only a consented population will likely bias the study results towards infants born to women who are stable on MOUD and miss a high-risk sub-population of infants with NOWS. This could be particularly problematic in this trial where there may be variation in the response to treatment. Each study site will use the assigned approach to care for all infants with NOWS throughout the trial. Therefore, obtaining consent would not alter the care approach used for the infant. Thus, the benefits afforded by using a waiver of consent outweigh the risks to the infant receiving the same treatment.
4. Whenever appropriate the study team will provide participants with additional pertinent information after participation.
  Throughout the study, the site study team will provide participants with additional pertinent information when appropriate. The site study team will (a) give to the caregiver(s) of all infants with NOWS who are cared for at the study site throughout the study and/or (b) post (as an enlarged copy) in appropriate location(s) readily visible to caregivers. This will fulfill the suggested framework [25] of participants being “provided with a detailed description of the study interventions to which their cluster has been randomized.”

Additional consent provisions for collection and use of participant data for neurodevelopmental follow-up and biological specimens may be added.

### Records retention

For NIH, grantees must retain records for a period of 3 years from the date of Federal Financial Report (FFR) submission.

## Statistical considerations and analytical plans

### Overview

This section briefly describes the analysis plan that will be followed for this study. A statistical analysis plan (SAP) will also be developed and finalized prior to database lock. The SAP will include a detailed description of each analysis and will precede any analyses described in this section.

### Analysis populations

The following analysis populations will include all randomized study sites that enrolled participants, regardless of the degree of adherence to the study interventions.

Intention-to-treat population (ITT): All enrolled participants at a study site during the study. Each participant will be analyzed according to the study intervention that the study site was assigned at the time the participant was born.

Intention-to-treat population—pharmacologically treated (ITT-PT): All participants in the ITT population who were pharmacologically treated at a study site during the study. Each participant will be analyzed according to the study intervention that the study site was assigned at the time the participant was born.

Intention-to-treat population—pharmacologically treated with symptom-based dosing approach (ITT-PT1): All participants included in the ITT-PT population who were pharmacologically treated using a symptom-based dosing approach.

Intention-to-treat population—pharmacologically treated with scheduled opioid taper approach (ITT-PT2): All participants included in the ITT-PT population who were pharmacologically treated using a scheduled opioid taper approach.

### Endpoints

Table 3 provides a summary of the endpoints that will be used to address the primary and secondary objectives.

**Table 3:** Objectives and endpoints

Objective	Analysis population	Endpoint
Primary objective and secondary objective #1	ITT	Number of days from birth until the participant meets criteria for medically ready for discharge. A participant is considered medically ready for discharge when they are discharged by the medical provider or when they meet both of the following criteria: • ≥ 96 h of age • ≥ 48 h since last dose of opioid treatment (i.e., morphine, methadone, or buprenorphine) In participants who meet withdrawal criteria prior to meeting the criteria for medically ready for discharge, this endpoint will be defined by the number of days from birth until the participant is withdrawn from the study
Secondary objective #2	ITT	Receipt of pharmacologic treatment, defined as having at least one dose of any amount of morphine, methadone, or buprenorphine (yes/no)
Secondary objective #3	ITT	Number of days from birth until hospital discharge. In participants who meet withdrawal criteria prior to hospital discharge, this endpoint will be defined by the number of days from birth until the participant is withdrawn from the study
Secondary objective #4	ITT	Inpatient composite safety outcome (present/absent), defined as the occurrence of the following between birth and hospital discharge: • Seizures • Excessive weight loss more than 15% from birthweight Inpatient composite critical safety outcome (present/absent), defined as the occurrence of the following between birth and hospital discharge: • Non-accidental trauma • Death Outpatient composite safety outcome (present/absent), defined as the occurrence of the following between hospital discharge and 3 months of age: • Acute/urgent care or emergency room visits • Hospital readmissions Outpatient composite critical safety outcome (present/absent), defined as the occurrence of the following between hospital discharge and 3 months of age: • Non-accidental trauma • Death
Secondary objective #5	ITT-PT (stratified by type of medication)	Number of doses of primary medication administered (count)
Secondary objective #6	ITT-PT	Receipt of secondary medications, defined as having at least one dose of any amount of morphine, methadone, or buprenorphine as a secondary medication (yes/no)
Secondary objective #7	ITT-PT1	Transitioned to an opioid taper due to persistent signs of withdrawal (yes/no)
Secondary objective #8	ITT-PT2	Cessation of opioid treatment due to excessive sedation (yes/no)
Secondary objective #9	ITT-PT	Inpatient composite safety outcome (present/absent), defined as the occurrence of the following between birth and hospital discharge: • Seizures • Excessive weight loss more than 15% from birthweight Inpatient composite critical safety outcome (present/absent), defined as the occurrence of the following between birth and hospital discharge: • Non-accidental trauma • Death Outpatient composite safety outcome (present/absent), defined as the occurrence of the following between hospital discharge and 3 months of age: • Acute/urgent care or emergency room visits • Hospital readmissions Outpatient composite critical safety outcome (present/absent), defined as the occurrence of the following between hospital discharge and 3 months of age: • Non-accidental trauma • Death

### Measures to minimize bias

The following measures will be taken to minimize bias in this study:

- Due to the nature of the study interventions, site-specific effects may arise. Consequently, a cluster crossover study design was selected because each study site will administer both study interventions at randomly selected times, thereby allowing for within-site estimates of the intervention effect.
- The assessment and management approach (FNAST or ESC) used at each study site may substantially affect the outcomes of interest. To address potential confounding, each study site will be required to use the same assessment and management approach for the duration of the study. Additionally, the randomization scheme will be stratified by the assessment and management approach to ensure that the percentage of study sites using each assessment and management approach is similar between the two sequences.

### Analysis plan

All statistical analyses will be performed by DCC biostatisticians. Data will be monitored for accuracy and completeness throughout the study. Analyses will only be performed after the database has been locked.

For descriptive summaries of study data, the following will be presented:

- Nominal/categorical measures will be summarized using frequencies and percentages.
- Interval or ratio scale measures will be summarized using means, standard deviations, medians, 95% confidence intervals (CIs), 25th and 75th percentiles, and ranges.
- Ordinal measures will be summarized depending on the number of levels. An ordinal measure with five levels or fewer will be summarized as a nominal measure. An ordinal measure with more than five levels will be summarized as an interval or ratio scale measure.

Unless otherwise specified, each objective will be addressed by fitting a generalized linear mixed-effects model (GLMM) specified with a distribution and link function appropriate to each participant-level outcome in infants with NOWS who are assessed and managed with FNAST and ESC separately. Each model will also include the following fixed effects:

- Dosing approach (symptom-based dosing approach or scheduled opioid taper approach)
- Study period in which participant was born (study period 1 or 2)

Random effects for site will also be included in the model to account for within- and between-period intra-cluster correlation. The form of the random effect for site will be evaluated by comparing information criteria across the models with different forms and selecting the form that produces the best fit. Small sample corrections will also be employed to account for the small number of sites that are randomized.

Because it is of clinical interest to separately evaluate all objectives in infants with NOWS who are assessed and managed with FNAST and ESC separately, no adjustment for multiple comparisons will be made.

#### Primary analysis of primary objective

Using the model outlined in the Analysis plan section, the number of days from birth until the participant meets criteria for medically ready for discharge will be analyzed in all infants in the ITT population who are assessed and managed with ESC using a GLMM with a negative binomial distribution and log link function. This model will be used to estimate the following quantities (and corresponding 95% CIs):

- Adjusted mean number of days until medically ready for discharge in infants managed for NOWS with a symptom-based dosing approach
- Adjusted mean number of days until medically ready for discharge in infants managed for NOWS with a scheduled opioid taper approach
- Difference in adjusted mean number of days until medically ready for discharge between infants managed for NOWS with a symptom-based dosing approach and infants who managed for NOWS with a scheduled opioid taper approach
- Adjusted relative rate (RR) of days until medically ready for discharge comparing infants managed for NOWS with a symptom-based dosing approach to infants managed for NOWS with a scheduled opioid taper approach

#### Supportive analyses of the primary objective

To investigate if the intervention effect (i.e., symptom-based dosing approach versus scheduled opioid taper approach) is modified by the study period in which the participant was born (study period 1 or 2), a two-way interaction between dosing approach and study period will be added to the model described in the Primary analysis of primary objective section. Further details regarding the interpretation of the fitted model will be provided in the SAP.

#### Analyses of secondary objectives

Using the model outlined in the Analysis plan section, Table 4 provides a summary of the endpoint, analysis population, statistical model, and resulting estimate for each of the secondary objectives.

**Table 4:** Statistical approach to be used for secondary objectives

Objective	Endpoint	Analysis population	Model	Estimate
Secondary objective #1	Number of days from birth until the participant meets criteria for medically ready for discharge	ITT (FNAST infants only)	GLMM • Distribution: negative binomial • Link function: log	Similar to the Primary analysis of primary objective section
Secondary objective #2	Receipt of pharmacologic treatment	ITT	GLMM • Distribution: binary • Link function: log	Adjusted risk of receipt of pharmacologic treatment for NOWS in infants managed with a symptom-based dosing approach Adjusted risk of receipt of pharmacologic treatment for NOWS in infants managed with a scheduled opioid taper approach Adjusted relative risk of receipt of pharmacologic treatment for NOWS between infants managed with a symptom-based dosing approach to infants managed with a scheduled opioid taper approach
Secondary objective #3	Number of days from birth until hospital discharge	ITT	GLMM • Distribution: negative binomial • Link function: log	Adjusted mean number of days until hospital discharge in infants managed with a symptom-based dosing approach Adjusted mean number of days until hospital discharge in infants managed with a scheduled opioid taper approach Difference in the adjusted mean number of days until hospital discharge between infants managed with a symptom-based dosing approach and infants managed with a scheduled opioid taper approach Adjusted RR of days until hospital discharge between infants managed with a symptom-based dosing approach to infants managed with a scheduled opioid taper approach
Secondary objective #4	• Inpatient composite safety outcome • Inpatient critical safety outcome • Outpatient composite safety outcome • Outpatient critical safety outcome	ITT	Descriptive statistics	Percentage of infants who met each endpoint will be summarized by study intervention
Secondary objective #5	Number of doses of primary medication administered	ITT-PT (stratified by type of medication)	GLMM • Distribution: negative binomial • Link function: log	Adjusted RR of doses of primary medication administered between infants who were pharmacologically treated with a symptom-based dosing approach to infants who were pharmacologically treated with a scheduled opioid taper approach
Secondary objective #6	Receipt of secondary medications	ITT-PT	GLMM • Distribution: binary • Link function: log	Adjusted relative risk of receipt of secondary medication between infants who were pharmacologically treated with a symptom-based dosing approach to infants who were pharmacologically treated with a scheduled opioid taper approach
Secondary objective #7	Transitioned to an opioid taper due to persistent signs of withdrawal	ITT-PT1	Descriptive statistics	Percentage of infants who were initially pharmacologically treated with a symptom-based dosing approach but transitioned to an opioid taper due to persistent signs of withdrawal
Secondary objective #8	Cessation of opioid treatment due to excessive sedation	ITT-PT2	Descriptive statistics	Percentage of infants who were initially pharmacologically treated with a scheduled opioid taper approach but stopped treatment due to excessive sedation
Secondary objective #9	• Inpatient composite safety outcome • Inpatient critical safety outcome • Outpatient composite safety outcome • Outpatient critical safety outcome	ITT-PT	Descriptive statistics	Percentage of infants who were pharmacologically treated and who met each endpoint will be summarized by study intervention

Similar to the sub-group analyses performed for the primary objective outlined in the Primary analysis of primary objective section, sub-group analyses will also be conducted for each secondary endpoint to determine if the intervention effect within each assessment and management approach is modified by the study period in which the participant was born.

#### Descriptive analyses

Baseline characteristics will be compared between the following groups using descriptive statistics:

- Within the ITT population:
  - ◦ Participants managed for NOWS using the symptom-based dosing approach and participants managed for NOWS using the scheduled opioid taper approach
  - ◦ Participants included in the ITT-PT population and participants not included in the ITT-PT population
- Within the ITT-PT population:
  - ◦ Participants who were pharmacologically treated using the symptom-based dosing approach and participants who were pharmacologically treated using the scheduled opioid taper approach

Each nominal/categorical variable will be summarized within each group and overall using a frequency and a percentage. The distribution of each nominal/categorical variable will be compared between the groups using a Pearson’s chi-squared test or Fisher’s exact test (as appropriate). Interval or ratio scale variables will be summarized within each group and overall using a mean, standard deviation, median, and inter-quartile range. The distribution of each interval or ratio scale variable will be compared between the groups using a Wilcoxon rank sum test. All tests will be performed using a two-sided significance level of 0.05. No adjustment for multiple comparisons will be performed.

### Interim analyses

#### Interim analysis of efficacy data

Using the approach outlined in the Primary analysis of primary objective section, one interim analysis testing the efficacy of the study intervention on the primary endpoint will be performed when the assessment of the primary endpoint is completed in approximately 50% of enrolled infants with NOWS. To account for multiple tests conducted at the interim and final analyses, the nominal significance level that will be used at the time of the interim and final analyses will be determined using O’Brien-Fleming stopping boundaries via the Lan-DeMets approach. Further details will be provided in the SAP.

#### Interim analysis of safety data

Summaries of safety data will be reviewed every 3–6 months by the DSMB. Additionally, summaries of safety data will also be included with the results of the interim analysis outlined in the Interim analysis of efficacy data section.

#### Futility analysis

An interim analysis for futility will not be performed.

### Statistical hypotheses

All objectives in this study will be addressed using two-sided hypothesis tests applied to the relevant analysis population and endpoint. For example, the null (H_0_) and alternative (H_A_) statistical hypotheses for the primary objective and secondary objective #1 are as follows:

- H_0_: The length of time from birth until medically ready for discharge in infants managed for NOWS with a symptom-based dosing approach is equal to infants managed for NOWS with a scheduled opioid taper approach.
- H_A_: The length of time from birth until medically ready for discharge in infants managed for NOWS with a symptom-based dosing approach is not equal to infants managed for NOWS with a scheduled opioid taper approach.

This hypothesis will be separately tested in two populations:

1. Infants who were assessed and managed with ESC (primary objective)
2. Infants who were assessed and managed with FNAST (secondary objective #1)

### Sample size considerations

The primary objective will be addressed by separately testing the intervention effect in infants who were assessed and managed with ESC. Secondary objective #1 will be addressed by separately testing the intervention effect in infants who were assessed and managed with FNAST. Consequently, sample size calculations have been provided for both groups.

Given the population and study interventions under study, sample size was selected by defining a clinically meaningful effect size of a 15%–25% reduction in time to medically ready for discharge when comparing this endpoint in infants with NOWS who are assessed and managed with ESC and who are treated with a symptom-based dosing approach to infants with NOWS who are treated with a scheduled opioid taper approach. To ensure that the study is conducted in the same time frame across the 16 ESC and 8 FNAST sites, a similar sample size will also be used for FNAST sites, acknowledging that a clinically meaningful effect size of 15%–25% may not be detectable with sufficient statistical power due to the smaller number of FNAST sites selected for this study.

All sample size calculations were performed using the Shiny CRT Calculator: Power and Sample Size for Cluster Randomized Trials [26] with the following options:

- Sampling structure: cross-sectional sample
- Correlation structure: exchangeable
- Allowance for varying cluster sizes: no
- Outcome type: count
- Normal approximation: *T*-distribution

Note that using data obtained from INFORM NOW during the periods of October 1, 2019–December 31, 2019, and July 1, 2021–March 31, 2022, we observed that:

- 349 infants met the eligibility criteria outlined in the Participant selection section and were assessed and managed with FNAST across 13 sites.
  - ◦ The mean time to medically ready for discharge was 16.35 days (95% CI: 12.97, 20.61) such that the estimated intra-class correlation coefficient was 0.1629 and the estimated over-dispersion parameter was 8.40.
  - ◦ 78.8% of participants received pharmacologic treatment.
- 133 infants met the eligibility criteria outlined in the Participant selection section and were assessed and managed with ESC across 8 sites.
  - ◦ The mean time to medically ready for discharge was 10.46 days (95% CI: 8.02, 13.63) such that the estimated intra-class correlation coefficient was 0.1119 and the estimated over-dispersion parameter was 5.46.
  - ◦ 39.1% of participants received pharmacologic treatment.

Assuming that the participants in OPTimize NOW are similar to those observed in INFORM NOW, sample size calculations of the primary endpoint given by time to medically ready for discharge were conducted under the following assumptions:

- In infants who were assessed and managed with FNAST:
  - ◦ The mean time to medically ready for discharge is approximately 16.50 in infants who are treated with a scheduled opioid taper approach.
  - ◦ Intra-class correlation coefficient in either arm is 0.15.
  - ◦ Over-dispersion parameter in either arm is 8.4.
- In infants who were assessed and managed with ESC:
  - ◦ The mean time to medically ready for discharge is approximately 10.50 in infants who are treated with a scheduled opioid taper approach.
  - ◦ Intra-class correlation coefficient in either arm is 0.15.
  - ◦ Over-dispersion parameter either arm is 5.5.

Under these assumptions, Table 5 shows that effect sizes that can be detected with 80% or 90% power in infants who are assessed and managed with ESC or FNAST using a total sample size of 480 participants (320 infants who are assessed and managed with ESC; 160 infants who are assessed and managed with FNAST) and assuming a two-sided significance level of 0.05. Specifically:

- In infants who are assessed and managed with ESC, a sample size of 320 will allow us to detect a relative rate in time to medically ready for discharge between infants who are treated with a symptom-based dosing approach versus infants who are treated with a scheduled opioid taper approach of 0.79 with 80% power and 0.76 with 90% power.
- In infants who are assessed and managed with FNAST, a sample size of 160 will allow us to detect a relative rate in time to medically ready for discharge between infants who are treated with a symptom-based dosing approach versus infants who are treated with a scheduled opioid taper approach of 0.71 with 80% power and 0.67 with 90% power.

**Table 5:** Sample size calculations

Treatment approach	Number of sites per sequence	Number of infants with NOWS per site per period	Number of infants with NOWS	Power	Time to medically ready for discharge		Relative rate of days to medically ready for discharge between arms
					Scheduled opioid taper	Symptoms-based dosing	
ESC	8	10	320	80%	10.50	8.3	0.79
				90%		8.0	0.76
FNAST	4	10	160	80%	16.50	11.7	0.71
				90%		11.0	0.67

If 2 participants are accrued per site per month across the 24 sites, a total sample size of 480 participants would require 5 months per period. The duration of each period is expected to be 5 months; this length may need to be modified to ensure sample size is met in Finnegan and ESC groups. Additionally, due to variation in enrollment across the sites and that enrollment in each period will end at the same time across all sites, the number of infants enrolled in the study may be higher than 480.

## Dissemination of results

### Data sharing

Data collected for this study will be analyzed and stored at the DCC, RTI International. NIH Helping to End Addiction Long-term Initiative (HEAL) and NICHD requirements for data sharing will apply. NIH has had a long-standing policy to share and make available to the public the results and accomplishments of the activities that it funds. In accordance with these policies (available at HEAL Public Access and Data Sharing | NIH HEAL Initiative), we will share de-identified data collected for the study. After the study is completed, the de-identified, archived data will be transmitted to a NIH HEAL supported repository, for use by other researchers including those outside of the study.

Public-use datasets will comply with NIH and its agencies requirements for sharing data through the creation of public-use datasets. The following procedures ensure that publicly released data satisfy all Health Insurance Portability and Accountability Act of 1996 (HIPAA) and any related requirements for protecting participant identity, including:

- Replacing the unique participant identification variable in the original datasets with a new unique participant identifier produced by a random number generator.
- Removing or recoding other distinguishing parameters, such as dates, study site identification numbers, and hospitals where medical procedures were performed. For example, dates can be recoded relative to a participant-specific reference point, and specific locations can be replaced by more general geographic codes.
- Combining subgroups with low frequencies or truncating distributions to ensure that there are a minimum number of participants for each category or value within each gender/ethnicity cell.

Appropriate documentation of raw datasets and edited analysis datasets are provided, including annotated CRFs, format files for variables, and data dictionaries. The data is typically provided in Statistical Analysis Software (SAS) datasets, with export files and documentation in Portable Document Format (PDF) format.

### Publication policy

Following completion of the study, investigators will publish the results of this research in a scientific journal with NICHD clearance. The Publication Committee will adhere to the trials registration policy adopted by the International Committee of Medical Journal Editors member journal.

## Conflict of interest disclosure

### Financial conflicts of interest of the institutions and investigators

The study investigators will have no financial conflicts of interest (FCOIs) related to the study outlined in this protocol.

### Plan for managing identified financial conflicts of interest

Any potential or perceived conflicts of interest, including FCOIs, per Title 42, Code of Federal Regulations, Part 50, Subpart F (50.604 Responsibilities of institutions regarding investigator FCOIs), as amended, requires institutional officials (and all subrecipients) to notify the grants officer of any FCOIs prior to expenditure of any funds and within 60 days of any subsequently identified FCOI. Institutional officials should also notify the National Institute of Child Health and Human Development at the same time regarding the COI management plan following institutional guidelines of each participating center.

## Trial status and declarations

### Trial status

Protocol version 5, December 5, 2024.

Enrollment began on March 25, 2024. Anticipated end of enrollment April, 2025. Follow-up (3 months) will be completed (estimated) July, 2025.

## Data Availability

This study will comply with the NIH Data Sharing Policy and Policy on the Dissemination of NIH-Funded Clinical Trial Information. Non-proprietary case report forms will be available on the NICHD Data and Specimen Hub.

## Abbreviations

AAP: American Academy of Pediatrics
AE: Adverse event
AESI: Adverse event of special interest
CI: Confidence interval
CRF: Case report form
DCC: Data coordinating center
DSMB: Data and Safety Monitoring Board
EDC: Electronic data capture
ESC: Eat, Sleep, Console
FNAST: Finnegan Neonatal Abstinence Scoring Tool
GCP: Good Clinical Practice
GLMM: Generalized linear mixed-effects model
HEAL: Helping to End Addiction Long-term Initiative
HELP for NOWS: HEAL Evaluation of Limited Pharmacotherapies for Neonatal Opioid Withdrawal Syndrome
HIPAA: Health Insurance Portability and Accountability Act of 1996
ICH: International Council for Harmonization
ITT: Intention-to-treat
ITT-PT: Intention-to-treat—pharmacologically treated
ITT-PT1: Intention-to-treat population—pharmacologically treated with symptom-based dosing approach
ITT-PT2: Intention-to-treat population—pharmacologically treated with scheduled opioid taper approach
MOUD: Medications for opioid use disorder
NICHD: National Institute of Child Health and Human Development
NIH: National Institutes of Health
NOWS: Neonatal opioid withdrawal syndrome
PDF: Portable Document Format
PI: Principal investigator
PRN: *pro re nata*
QC: Quality control
QI: Quality improvement
RR: Relative rate
RUCA: Rural-urban commuting area
SAE: Serious adverse event
SAP: Statistical analysis plan
SAS: Statistical Analysis Software
SC: Steering Committee
sIRB: Single Institutional Review Board
